# Associations and prognostic implications of Eastern Cooperative Oncology Group performance status and tumoral *LINE-1* methylation status in stage III colon cancer patients

**DOI:** 10.1186/s13148-016-0203-8

**Published:** 2016-04-05

**Authors:** Duo Chen, Xianyu Wen, Young Seok Song, Ye-Young Rhee, Tae Hun Lee, Nam Yun Cho, Sae-Won Han, Tae-You Kim, Gyeong Hoon Kang

**Affiliations:** Key Laboratory of Carcinogenesis and Translational Research (Ministry of Education), Department of Cancer Epidemiology, Peking University Cancer Hospital and Institute, Beijing, China; Department of Pathology, Seoul National University College of Medicine, Seoul, Korea; Laboratory of Epigenetics, Cancer Research Institute, Seoul National University College of Medicine, Seoul, Korea; Division of Oncology, Department of Internal Medicine, Seoul National University College of Medicine, Seoul, Korea

**Keywords:** Adjuvant therapy, Colorectal cancer, ECOG, FOLFOX, *LINE-1*, Methylation, Prognosis

## Abstract

**Background:**

Low methylation status of *LINE-1* in tumors is associated with poor survival in patients with colon cancer. Eastern Cooperative Oncology Group performance status (ECOG-PS) is a method to assess the functional status of a patient. We retrospectively evaluated the relationship between ECOG-PS and *LINE-1* methylation in colorectal cancers (CRCs) and their prognostic impact in CRC or colon cancer patients receiving adjuvant 5-fluorouracil/leucovorin/oxaliplatin (FOLFOX).

**Results:**

*LINE-1* methylation and microsatellite instability were analyzed in stage III or high-risk stage II CRCs (*n* = 336). *LINE-1* methylation levels were correlated with clinicopathological features, including PS and recurrence-free survival (RFS). The association between the tumoral *LINE-1* methylation level and PS was observed (OR = 2.56, *P* < 0.001). Differences in *LINE-1* methylation levels in cancer tissue between the PS 0 and 1 groups were significant in patients older than 60 years (*P* = 0.001), the overweight body mass index group (*P =* 0.005), and the stage III disease group (*P* = 0.008). Prognostic significances of *LINE-1* methylation status or combined PS and *LINE-1* methylation statuses were identified in stage III colon cancers, not in stage III and high-risk stage II CRCs. Low *LINE-1* methylation status was closely associated with a shorter RFS time. The difference between PS(0)/*LINE-1*(high) and PS(≥1)/*LINE-1*(low) was significant, which suggests that colon cancer patients with concurrent PS(≥1)/*LINE-1* (low) have a higher recurrence rate.

**Conclusions:**

PS was associated with *LINE-1* methylation in CRC tissue. *LINE-1* methylation was associated with RFS in stage III colon cancer patients who were treated with adjuvant FOLFOX chemotherapy. Combined PS and *LINE-1* methylation status might serve as a useful predictor of cancer recurrence.

**Electronic supplementary material:**

The online version of this article (doi:10.1186/s13148-016-0203-8) contains supplementary material, which is available to authorized users.

## Background

Colorectal cancer (CRC) is one of the most common cancers and a leading cause of cancer death globally [1]. Despite a declined CRC mortality in developed countries, the incidence and mortality are increasing in East Asian populations, including Korea [2, 3]. Adjuvant chemotherapy significantly decreases mortality in colon cancer, and 5-fluorouracil (5-FU), leucovorin, and oxaliplatin (FOLFOX) are the current standard of care for patients with stage III colon cancer after surgery [4, 5]. FOLFOX is also frequently used to treat stage II colon cancer with high-risk features, such as T4 tumors or lymphovascular invasion.

CRC is a multifactorial disease which arises due to the accumulation of genetical and epigenetical alterations. Epigenetic changes are potential factors contributing to the carcinogenesis of colorectal cancer (CRC). Genome-wide hypomethylation is an alternative mechanism for genomic instability, which facilitates tumor progression [6]. Methylation levels of repetitive transposable DNA elements are a useful surrogate marker for global genomic methylation status because repetitive DNA elements reside in intergenic or intronic regions of the genome at extremely high frequencies, and CpG sites located within repetitive DNA elements are usually methylated [7]. Long interspersed nucleotide element-1 (*LINE-1*) is a major constituent of repetitive transposable DNA elements, and it constitutes approximately 17 % of the human genome [7, 8]. *LINE-1* is usually methylated in normal cells, which maintains transcriptional inactivation and inhibits retrotransposition [9]. Hypomethylation of *LINE-1* is a common finding in various tissue types of human cancer, and recent meta-analysis results have shown that *LINE-1* methylation levels are significantly lower in cancer tissues than in paired normal tissues, and especially in CRC and gastric cancer [10–17]. In some tissue types of human cancer, including CRC, tumoral *LINE-1* hypomethylation is associated with a poor clinical outcome [14, 15, 18–21].

The Eastern Cooperative Oncology Group performance status (ECOG-PS) is a global assessment of a cancer patient’s actual level of function and self-care ability [22, 23]. ECOG-PS is strongly associated with prognosis in various tissue types of cancer, including CRC [24–31]. Poor PS (PS score ≥2) is a strong predictor of poor prognosis in patients with metastatic CRC and tends to be associated with poor prognosis in patients with non-metastatic CRC [32, 33]. Although increasing stages of tumor depth or nodal metastasis tend to be accompanied by functional declines of the patients, little is known regarding the relationship between PS and cancer biology, including molecular phenotypes. A couple of recent studies have shown that a decrease in tumoral DNA methylation content leads to endogenous retrovirus activation and subsequent overexpression of interferon-pathway genes [34, 35]. Proinflammatory signals from tumor cells or aberrant host response to tumor presence might affect PS of the cancer patient [36]. Thus, it can be speculated that PS might play a confounding role in the effect of tumoral *LINE-1* methylation status as a predictor of recurrence. Recently, Lou et al.’s study has demonstrated that low *LINE-1* methylation status is an independent risk factor for recurrence in stage III colon cancer patients treated with adjuvant FOLFOX [37]. However, the study of Lou et al. did not consider PS as a potential predictor of recurrence. In this study, we analyzed adjuvant FOLFOX-treated stage III or high-risk stage II CRCs for their methylation levels of *LINE-1* using pyrosequencing methylation assay and correlated tumoral *LINE-1* methylation status with multiple clinicopathological parameters, including PS and recurrence-free survival of the patient.

## Results

### Distribution of clinicopathological parameters in different PS groups

A total of 336 patients were included. Clinicopathological parameters of the patients are described in Table 1. PS ≥1 was present in 170 (50.5 %) patients (169 patients with PS 1 and one patient with PS 2). The majority of patients were men (*n* = 211, 62.8 %), and the average age was 58.9 years (median 60, range 29–78 years). The tumor location was cecum in 9 patients, ascending colon in 75, transverse in 21, descending in 22, sigmoid in 189, and rectum in 20, including 104 right colon carcinomas and 212 left colon carcinomas. T stages 1/2/3/4 were 8/30/255/43 in patients, respectively, and N stages 0/1/2 were 49/203/84 in patients, respectively. Forty-nine patients had high-risk stage II disease (IIA, 33; IIB, 12; IIC, 4), and 287 patients had stage III disease (IIIA, 31; IIIB, 195; IIIC, 61). A total of 165 patients received FOLFOX-4, and 171 patients received modified FOLFOX-6. *KRAS* and *BRAF* mutations were found in 26.0 and 2.8 % of CRCs, respectively. Between the two different PS groups, the distributions of N stage (*P =* 0.032), microsatellite instability (MSI) status (*P =* 0.035), and the ratio of tumor to normal lymph node *LINE-1* methylation level (TNR) (*P* = 0.002) were different, but the other parameters were not (Table 1).

**Table 1:** Baseline characteristics of the epidemiological and clinical variables of the study population

Parameters	ECOG performance status					
	Case *N* (%)	0 *N* (%)	≥1 *N* (%)	*P* value^a^	*P* value^b^	OR
Gender						
Female	125 (37.2)	64 (38.6)	61 (35.9)			
Male	211 (62.8)	102 (61.4)	109 (64.1)	0.612	0.699	1.10
Age (median)						
<60 years	165 (49.1)	90 (54.2)	75 (44.1)			
≥60 years	171 (50.9)	76 (45.8)	95 (55.9)	0.064	0.126	1.37
BMI						
Normal	135 (40.2)	74 (44.6)	61 (35.9)			
Overweight	201 (59.8)	92 (55.4)	109 (64.1)	0.104	0.234	1.33
Tumor location						
Proximal	105 (31.3)	54 (32.5)	51 (30.0)			
Distal	231 (68.7)	112 (67.5)	119 (70.0)	0.617	0.885	1.11
Pathology						
Non-mucinous carcinoma	321 (95.5)	160 (96.4)	161 (94.7)			
Mucinous adenocarcinoma	15 (4.5)	6 (3.6)	9 (5.3)	0.599^c^	0.965	0.82
Differentiation						
Low grade	311 (92.6)	156 (94.0)	155 (91.2)			
High grade	25 (7.4)	10 (6.0)	15 (8.8)	0.328	0.185	2.51
T stage						
T1, 2, 3	293 (87.2)	147 (88.6)	146 (85.9)	0.464	0.568	1.08
T4	43 (12.8)	19 (11.4)	24 (14.1)			
N stage						
N 0, 1	252 (75.0)	133 (80.2)	119 (70.0)	0.032	0.088	1.67
N 2	84 (25.0)	33 (19.9)	51 (30.0)			
Microsatellite status						
MSS + MSI-L	313 (93.1)	149 (89.8)	164 (96.5)	0.035	0.024	0.30
MSI-H	21 (6.3)	15 (9.0)	6 (3.5)			
Missing	2 (0.6)	2 (1.2)	0			
*KRAS*						
Wild type	230 (68.5)	122 (73.5)	108 (63.5)	0.342	0.367	1.38
Mutant type	81 (24.1)	38 (22.9)	43 (25.3)			
Missing	25 (7.4)	6 (3.6)	19 (11.2)			
*BRAF*						
Wild type	316 (94.0)	159 (95.8)	157 (92.4 %)	0.333	0.502	0.56
Mutant type	9 (2.7)	6 (3.6)	3 (1.8)			
Missing	11 (3.3)	1 (0.6)	10 (5.9)			
TNR (median)						
<0.69	173 (51.5)	71 (42.8)	102 (60.0)	0.002	0.001	0.04
≥0.69	163 (48.5)	95 (57.2)	68 (40.0)			

*BMI* body mass index, *ECOG* Eastern Cooperative Oncology Group, *MSS* microsatellite stable, *MSI-L* microsatellite instability-low, *MSI-H* microsatellite instability-high, *TNR* tumor to normal ratio

^a^Chi-square test

^b^Unconditional logistic regression, adjusted for other selected covariates

^c^Fisher’s exact test

### Association between *LINE-1* methylation and clinicopathological parameters

To determine clinicopathological implications of the *LINE-1* methylation status, we analyzed *LINE-1* methylation in cancer and normal lymph node (LN) tissue samples. *LINE-1* methylation levels in cancer tissue samples ranged from 29.81 to 78.73 % (median, 52.64 %; standard deviation, 8.588 %), which was significantly lower than those of normal LN tissue samples which ranged from 48.50 to 89.90 % (median, 76.80 %; standard deviation, 4.138 %) (*P* < 0.001). The TNR ranged from 0.63 to 0.78 with median of 0.69 (standard deviation, 0.117).

Association between *LINE-1* methylation levels in cancer tissue samples and the PS of the patients were observed (OR = 2.56, *P* < 0.001). *LINE-1* methylation levels in normal LNs were also associated with body mass index (BMI) status of the patient (OR = 1.76, *P =* 0.015). No association was found between other selected parameters, such as MSI status, and *LINE-1* methylation levels of cancer or normal LN tissue samples (Table 2).

**Table 2:** The association between *LINE-1* methylation status and clinical variables of the study population (stage III and high-risk stage II CRCs)

Parameters	*LINE-1* methylation levels (low vs. high)^a^					
	Cancer sample (*n* = 336)			Normal lymph node sample (*n* = 336)		
	OR	(95 % C.I.)	*P* ^b^	OR	(95 % C.I.)	*P* ^b^
Gender						
(Male vs. female)	1.49	(0.921–2.406)	0.104	1.06	(0.670–1.677)	0.803
Age						
(≥60 years vs. <60 years)	1.20	(0.752–1.899)	0.450	0.95	(0.611–1.480)	0.823
BMI						
(Overweight vs. normal)	1.24	(0.775–1.996)	0.366	1.76	(1.118–2.765)	0.015
Tumor location						
(Distal vs. proximal)	0.81	(0.474–1.373)	0.429	1.26	(0.762–2.079)	0.370
Pathology						
(Mucinous vs. non-mucinous)	0.83	(0.196–3.521)	0.802	1.44	(0.349–5.926)	0.616
Differentiation						
(High grade vs. low grade)	0.37	(0.111–1.211)	0.100	0.68	(0.213–2.173)	0.515
T stage						
(T4 vs. T1, 2, 3)	0.81	(0.403–1.629)	0.555	0.68	(0.347–1.324)	0.255
N stage						
(N2 vs. N 0, 1)	1.24	(0.715–2.156)	0.443	1.15	(0.681–1.938)	0.602
Microsatellite status						
(MSI-H vs. MSS + MSI-L)	0.33	(0.106–1.008)	0.052	0.76	(0.283–2.026)	0.580
Performance status						
(PS ≥1 vs. PS = 0)	2.56	(1.601–4.102)	<0.001	1.12	(0.718–1.752)	0.615

*BMI* body mass index, *MSI-H* microsatellite instability, *MSS* microsatellite stable, *MSI-L* microsatellite instability-low, *PS* performance status

^a^Cut-off points of *LINE-1* methylation proportion were 54.62 % in cancer tissue samples and 77.00 % in normal lymph node samples

^b^Unconditional logistic regression, adjusted for other selected covariates

**Fig. 1:**
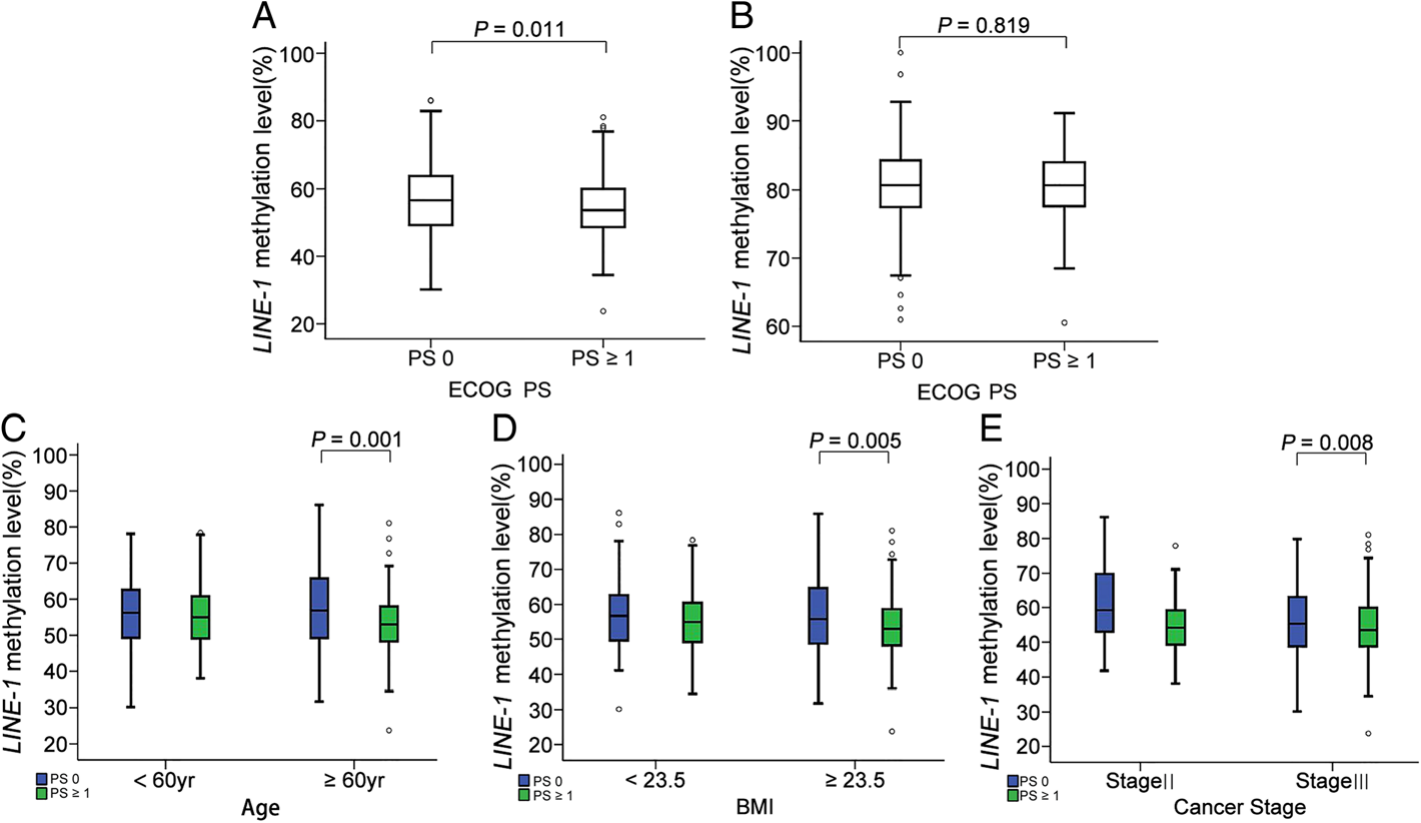
Comparison of *LINE-1* methylation levels between PS = 0 and PS ≥ 1 groups. **a**, **b** *LINE-1* methylation levels are significantly different between PS = 0 and PS ≥ 1 groups in cancer tissues (**a**) but not in normal lymph node samples (**b**). **c**–**e** Differences in *LINE-1* methylation levels in cancer tissues between two PS groups were significant in patients who were older than 60 years (**c**), had a BMI indicating overweight (**d**), or stage III disease (**e**)

*LINE-1* methylation levels were significantly lower in cancer tissue samples of patients with PS ≥ 1 than in those of patients with PS 0 (Fig. 1a). However, such a difference was not found in normal LN samples (Fig. 1b). The differences in *LINE-1* methylation in cancer tissues between PS 0 and PS ≥ 1 were significant in patients aged ≥60 years, overweight patients (BMI ≥23.5), and stage III cancers but not in patients aged <60 years, low BMI patients (BMI <23.5), and stage II cancers (Fig. 1c–e).

### PS status, *LINE-1* methylation status, and RFS

The median follow-up of the entire cohort at the time of data cut-off for the present analysis was 50.30 months, interquartile range (IQR) 46.68–53.92 months. At the last follow-up, a total of 41 disease-recurrence events (35 distant metastases and 6 local recurrences) were observed. The overall 4.5-year and 9-year recurrence-free survival (RFS) estimates were 87.50 and 83.00 %, respectively.

**Fig. 2:**
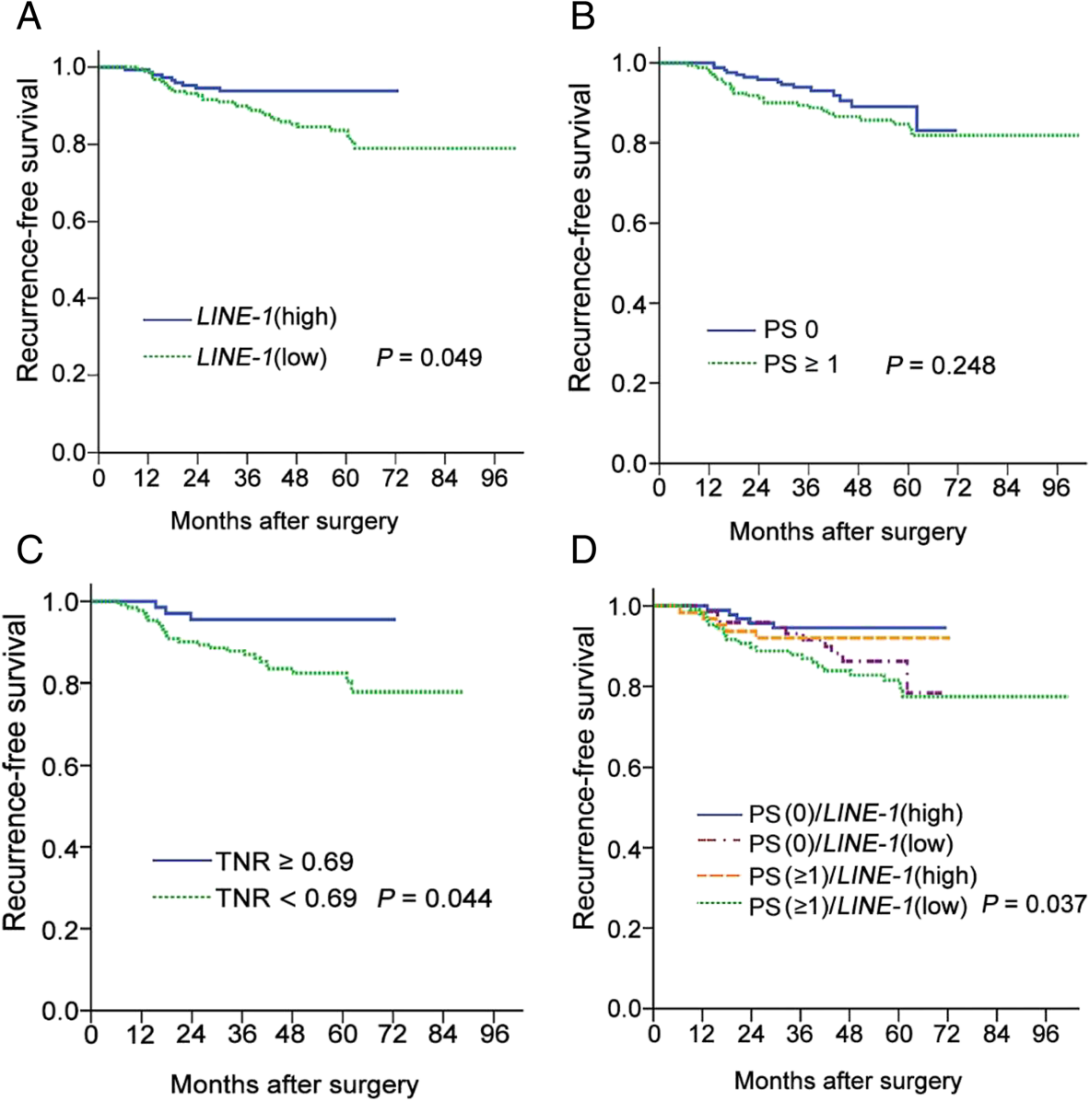
Survival analyses. **a** Low *LINE-1* methylation status was closely associated with shorter RFS times (*P* = 0.049). **b** There was no significant difference in recurrence-free survival according to the performance status (PS) score. **c** Survival analysis in patients with overweight BMI score according to the tumor to normal ratio (TNR) of *LINE-1* methylation level. **d** In survival analysis stratified by combinatory PS and *LINE-1* methylation statuses, recurrence-free survival was significantly different between PS(0)/*LINE-1*(high) and PS(1)/*LINE-1*(low) (*P* = 0.037)

*LINE-1* methylation was associated with RFS. The cut-off value for *LINE-1* methylation levels was determined as 54.62 % which was the median of *LINE-1* methylation levels in the PS 0 group. This median value was close to the value (54.12 %) which generated the maximum value of Youden index used in conjunction with the receiver operating characteristic curve analysis. Low *LINE-1* methylation status (<54.62 %) was closely associated with a shorter RFS time. The 4.5-year RFS was 93.9 % in the high *LINE-1* methylation patients, and it was 84.9 % in the low *LINE-1* methylation patients (log-rank test, *P* = 0.049) (Fig. 2a). There was no significant difference in RFS according to the PS: 4.5-year RFS 93.9 % in the PS 0 group and 85.7 % in the PS ≥1 (*P* = 0.248, Fig. 2b). However, multivariate Cox regression analysis revealed that *LINE-1* methylation status was not an independent prognostic parameter (Table 3).

**Table 3:** Impact of clinicopathological parameters, including performance status and LINE-1 methylation status, on cancer recurrence in stage III or high-risk stage II CRCs (*n* = 336), assessed by univariate and multivariate Cox regression analyses

Parameters	Univariate analysis			Multivariate analysis		
	HR	95 % C.I.	*P* ^a^	HR	95 % C.I.	*P* ^a^
Gender (male vs. female)	1.66	0.834–3.321	0.149			
Age (≥60 years vs. <60 years)	1.45	0.774–2.719	0.245			
BMI (overweight vs. normal)	1.29	0.674–2.454	0.445			
Tumor location (distal vs. proximal)	1.94	0.897–4.205	0.092			
Pathology (mucinous vs. non-mucinous)	1.29	0.311–5.343	0.726			
Differentiation (high grade vs. low grade)	1.15	0.353–3.718	0.821			
T stage (T4 vs. T1, 2, 3)	2.41	1.179–4.908	0.016	2.33	1.142–4.761	0.020
N stage (N 2 vs. N0, 1)	2.51	1.355–4.654	0.003	2.46	1.328–4.569	0.004
Microsatellite status (MSI-H vs. MSS + MSI-L)	1.24	0.381–4.002	0.725			
Performance status (PS ≥ 1 vs. PS = 0)	1.46	0.766–2.770	0.251			
*LINE-1* methylation (low vs. high)	2.15	0.984–4.701	0.055			

*BMI* body mass index, *MSS* microsatellite stable, *MSI-L* microsatellite instability-low, *MSI-H* microsatellite instability-high, *PS* performance status

^a^Cox proportional hazard regression model, adjusted for other selected covariates

We also performed survival analysis using the TNR value stratified by selected parameters such as age, gender, BMI, tumor location, T stage, N stage, and ECOG status. Low TNR value (<0.69) was closely associated with a shorter RFS time in the overweight patients (higher BMI score) (Fig. 2c). The 4.5-year RFS was 95.6 % in the high TNR value patients, and it was 83.8 % in the low TNR value patients (log-rank test, *P* = 0.044). The TNR value was not associated with other selected parameters.

When we counted RFS from the date of chemotherapy to the date of recurrence, Kaplan-Meier log-rank analysis showed survival curves similar to those using RFS calculated from the date of surgery to the date of recurrence by the Kaplan-Meier method with log-rank test (Additional file 1: Figure S1).

### Combinatory PS and *LINE-1* methylation statuses as a predictor of recurrence in the setting of adjuvant FOLFOX

When we performed survival analysis with combinatory PS and *LINE-1* methylation statuses, we found that the 4.5-year RFS was 94.5 % in the PS(0)/*LINE-1*(high) group (*n* = 93 patients), 91.5 % in the PS(0)/*LINE-1*(low) group (*n* = 73), 92.1 % in the PS(≥1)/*LINE-1*(high) group (*n* = 63), and 81.5 % in the PS(≥1)/*LINE-1*(low) group (*n* = 107). The difference was significant between PS(0)/*LINE-1*(high) and PS(≥1)/*LINE-1*(low) (pairwise pooled log-rank test, *P* = 0.037) (Fig. 2d). We did not perform further analyses of RFS in combination with *BRAF* and *KRAS* mutation statuses because of the low number of patients with this mutation.

Univariate analyses revealed that higher levels of T stage (HR = 2.41, 95 % C.I. = 1.179–4.908, *P =* 0.016), N stage (HR = 2.51, 95 % C.I. = 1.355–4.654, *P =* 0.003) and PS(≥1)/*LINE-1*(low) (HR = 2.72, 95 % C.I. = 1.013–7.325, *P =* 0.047) were associated with higher recurrence. Multivariable Cox regression analyses adjusting for gender, age, BMI, tumor location, histology, T stage, N stage, and microsatellite instability status revealed consistent statistical patterns and *P* values in T stage (HR = 2.60, 95 % C.I. = 1.251–5.413, *P =* 0.011) and N stage (HR = 2.78, 95 % C.I. = 1.429–5.393, *P =* 0.003), but PS(≥1)/*LINE-1*(low) status was not associated with RFS (HR = 2.27, 95 % C.I. = 0.839–6.168, *P =* 0.106) (Table 4).

**Table 4:** Univariate and multivariate Cox regression analyses for recurrence-free survival in stage III or high-risk stage II CRCs (*n* = 336)

Parameters	Univariate analysis			Multivariate analysis		
	HR	95 % C.I.	*P*	HR	95 % C.I.	*P* ^a^
T stage (T4 vs. T1, 2, 3)	2.41	1.179–4.908	0.016	2.36	1.153–4.818	0.019
N stage (N 2 vs. N0, 1)	2.51	1.355–4.654	0.003	2.43	1.309–4.513	0.005
Performance status—*LINE-1* methylation						
PS(0)/*LINE-1* (high)	Ref.			Ref.		
PS(0)/*LINE-1* (low)	2.02	0.684–5.936	0.203	2.07	0.704–6.105	0.185
PS(≥1)/*LINE-1* (high)	1.25	0.360–4.337	0.343	1.20	0.344–4.152	0.778
PS(≥1)/*LINE-1* (low)	2.72	1.013–7.325	0.047	2.59	0.966–6.964	0.059

*BMI* body mass index, *ECOG* Eastern Cooperative Oncology Group, *MSS* microsatellite stable, *MSI-L* microsatellite instability-low, *MSI-H* microsatellite instability-high, *PS* performance status

^a^Cox proportional hazard regression model, adjusted for other selected covariates

### Prognostic value of *LINE-1* methylation status alone or combined PS and *LINE-1* statuses in stage III colon cancers

Because high-risk stage II CRCs or stage III rectal cancers may differ from stage III colon cancers with respect to the beneficial effect from adjuvant FOLFOX vs. 5-FU/leucovorin, survival analysis was needed to be performed in stage III colon cancers with exclusion of rectal cancers and stage II colon cancers. The associations between *LINE-1* methylation levels with covariates investigated in the study population (stage III colon cancers only) (Table 5) were similar to those between *LINE-1* methylation levels with covariates investigated in the study population (stage III colon cancers plus high-risk stage II CRCs and stage III rectal cancers) (Table 2). Multivariate Cox regression analysis revealed that low *LINE-1* methylation status was associated with a shorter RFS time (Table 6). PS(≥1)/*LINE-1*(low) was independently associated with disease recurrence (HR = 5.06, 95 % C.I. = 1.142–22.377, *P* = 0.033) in multivariate analysis (Table 7).

**Table 5:** The association between *LINE-1* methylation status and clinicopathological parameters (stage III colon cancers only)

Parameters	*LINE-1* methylation levels (low vs.high)					
	Cancer sample (*n* = 268)			Normal LN sample (*n* = 268)		
	OR	(95 % C.I.)	*P* ^a^	OR	(95 % C.I.)	*P* ^a^
Gender (male vs. female)	1.63	(0.971–2.747)	0.065	1.11	(0.699–1.745)	0.670
Age (≥60 years vs. <60 years)	1.23	(0.742–2.053)	0.419	0.97	(0.624–1.508)	0.893
BMI (overweight vs. normal)	1.21	(0.711–2.044)	0.487	1.56	(0.991–2.444)	0.055
Tumor location (distal vs. proximal)	0.78	(0.438–1.383)	0.393	1.14	(0.694–1.887)	0.598
Pathology						
(Mucinous vs. non-mucinous)	0.77	(0.149–4.004)	0.759	1.52	(0.361–6.387)	0.569
Differentiation						
(High grade vs. low grade)	0.58	(0.154–2.185)	0.420	0.51	(0.154–1.687)	0.270
T stage (T4 vs. T1, 2, 3)	0.99	(0.417–2.359)	0.985	0.74	(0.381–1.447)	0.382
N stage (N2 vs. N0, 1)	1.07	(0.600–1.921)	0.812	1.21	(0.720–2.039)	0.470
Microsatellite status						
(MSI-H vs. MSS + MSI-L)	0.40	(0.118–1.388)	0.150	1.02	(0.380–2.728)	0.972
Performance status (PS ≥ 1 vs. PS = 0)	1.80	(1.081–3.004)	0.024	1.20	(0.770–1.871)	0.421

*BMI* body mass index, *MSS* microsatellite stable, *MSI-L* microsatellite instability-low, *MSI-H* microsatellite instability-high, *PS* performance status

^a^Unconditional logistic regression, adjusted for other selected covariates

**Table 6:** Univariate and multivariate Cox regression analyses for recurrence-free survival in stage III colon cancers (*n* = 268)

Parameters	Univariate analysis			Multivariate analysis		
	HR	95 % C.I.	*P* ^a^	HR	95 % C.I.	*P* ^a^
Gender (male vs. female)	1.96	0.881–4.367	0.099			
Age (≥60 years vs. <60 years)	1.54	0.775–3.161	0.234			
BMI (overweight vs. normal)	2.23	0.966–5.164	0.060			
Tumor location (distal vs. proximal)	2.32	0.955–5.637	0.063			
Pathology (mucinous vs. non-mucinous)	1.71	0.409–7.166	0.462			
Differentiation (high grade vs. low grade)	1.41	0.428–4.619	0.575			
T stage (T4 vs. T1, 2, 3)	2.97	1.284–6.865	0.011	2.50	1.065–5.854	0.035
N stage (N2 vs. N0, 1)	2.40	1.196–4.795	0.014	2.26	1.114–4.575	0.024
Microsatellite status (MSI-H vs. MSS + MSI-L)	1.06	0.254–4.451	0.933			
Performance status (PS ≥ 1 vs. PS 0)	1.63	0.782–3.394	0.192			
*LINE-1* methylation (low vs. high)	3.36	1.287–8.781	0.013	3.50	1.338–9.126	0.011

*BMI* body mass index, *MSS* microsatellite stable, *MSI-L* microsatellite instability-low, *MSI-H* microsatellite instability-high, *PS* performance status

^a^Cox proportional hazard regression model, adjusted for other selected covariates

**Table 7:** Univariate and multivariate Cox regression analyses for recurrence-free survival in stage III colon cancers only (*n* = 268)

Parameters	Univariate analysis			Multivariate analysis		
	HR	95 % C.I.	*P* ^a^	HR	95 % C.I.	*P* ^a^
T stage (T1, 2, 3 vs. T4)	2.97	1.284–6.865	0.011	2.97	1.284–6.865	0.036
N stage (N0, 1 vs. N2)	2.40	1.196–4.795	0.014	2.40	1.196–4.795	0.023
Performance status*—LINE-1* methylation						
PS(0)/*LINE-1* (high)	Ref.			Ref.		
PS(0)/*LINE-1* (low)	3.69	0.794–17.167	0.096	3.69	0.794–17.167	0.082
PS(≥1)/*LINE-1* (high)	1.78	0.297–10.678	0.528	1.78	0.297–10.678	0.528
PS(≥1)/*LINE-1* (low)	5.20	1.197–22.611	0.028	5.20	1.197–22.611	0.025

*PS* performance status

^a^Cox proportional hazard regression model, adjusted for other selected covariates

## Discussion

The present study has demonstrated that ECOG-PS was associated with *LINE-1* methylation in cancer tissue. The difference in *LINE-1* methylation in cancer tissue between PS 0 and PS ≥ 1 was significant in patients of older age, higher BMI score, or stage III disease. *LINE-1* methylation levels change with aging and disease progression, and our stratified analyses of *LINE-1* methylation levels adjusting for potential confounding suggested that low levels of *LINE-1* methylation had a closer relationship with poorer PS. To our knowledge, the present study is the first study to assess the association between PS statuses of CRC patients and *LINE-1* methylation levels of cancer tissue.

A previous study suggests that *LINE-1* methylation is a strong, independent predictor of recurrence-free survival in adjuvant FOLFOX-treated stage III colon cancers [32]. Our present study also showed that low *LINE-1* methylation status was closely associated with shorter RFS time. However, PS was not associated with RFS. This result was not consistent with previous studies showing that PS was predictive of survival in patients with locally advanced or metastatic carcinoma [38, 39]. There are several factors triggering this result. First, this result may be due to the small number of relapse events and the follow-up of RFS. The Kaplan-Meier analysis showed that the curves of PS 0 and PS ≥ 1 separated early, and most events happened before the median follow-up time, which is an inaccurate measurement of the median RFS. Another reason may be the limited PS subgroups in the study. PS was distributed into mainly two groups: PS 0 and PS 1, with only one patient having PS 2. The lack of patients with PS >1 limited the power to distinguish significant differences in RFS with different PS groups. Because the result was not significant in our condition, we performed statistical power analyses to decide the likelihood that the test can detect effects of a given size in this particular situation. The power analysis found that the overall 336 subjects (170 in PS ≥ 1 and 166 in PS 0) achieved a 56.93 % power at a 0.050 significance level to detect a difference in RFS between PS ≥ 1 and PS 0 (Additional file 2: Figure S2A). The sample size estimation suggested that a larger number should be included in further study if the relapse events are rare while the relapse-free proportion stays high during follow-up of the PS ≥ 1 group. The stronger power of the precision to provide reliable answers requires larger sample numbers (Additional file 2: Figure S2B).

Our results suggested that RFS was highest in CRCs with PS(0)/*LINE-1*(high) and lowest in CRCs with PS(≥1)/*LINE-1*(low). Therefore, we just found a significant difference between the two groups of CRCs in univariate analysis. This difference was not significant in multivariate analyses. This lack of significance may be affected by other parameters that are involved in multivariate analysis and the small number of patients in each PS/*LINE-1* groups after stratification. However, in stage III colon cancer patients with exclusion of rectal cancers and high-risk stage II colon cancers patients, not only *LINE-1* methylation status alone but also PS(≥1)/*LINE-1*(low) was independently associated with disease recurrence in multivariate analysis. Whether PS(≥1)/*LINE-1*(low) might identify a subset of patients with poor prognosis is required to be validated with a large-size samples of stage III colon cancers.

The TNR value is more appealing parameter compared with just methylation level of *LINE-*1 measured in cancer tissue samples. The value reflects methylation change in tumor compared with matched normal tissue and removes some artifacts inherent using FFPE samples and methylation analysis technology. It provides possible normalization of many confounding variables in the analysis technology. We found that low TNR value was closely associated with a shorter RFS time in the overweight patient.

Measurement of *LINE-1* methylation level in bisulfite-treated genomic DNA samples using MethyLight or pyrosequencing assay has been shown to correlate with genomic DNA methylation levels measured by liquid chromatography-mass spectrometry [40, 41], which provided a basis for use of *LINE-1* pyrosequencing methylation assay as a surrogate marker for genomic DNA methylation level. The precision and reliability of *LINE-1* pyrosequencing assay have been shown in Irahara et al.’s study which evaluated performance of pyrosequencing assay of *LINE-1* methylation levels in genomic DNA samples from formalin-fixed paraffin-embedded (FFPE) tissue samples, fresh-frozen tissue samples, and peripheral blood leukocytes [42]. Our study measured the same four CpG sites which Irahara et al.’s study assayed using pyrosequencing. However, Tournier et al. raised a doubt upon results of pyrosequencing methylation assay in FFPE tissue samples because they found discrepant methylation levels in paired fresh-frozen and FFPE tissue samples [43]. In our previous study [44], we analyzed paired fresh-frozen and FFPE tissue samples for their methylation level at the four CpG sites of *LINE-1* and found that FFPE tissue samples tended to show increased methylation levels at four CpG sites compared with fresh tissue samples although methylation levels of individual CpG sites showed strong correlations between paired fresh-frozen and FFPE tissue samples. The fold-increase was similar at CpG sites 2 and 3 but different from that of CpG site 1 or 4 [44], and thus, we determined to obtain average value of methylation levels at CpG sites 2 and 3 as representative value of *LINE-1* methylation levels in FFPE tissue samples.

The present study has several limitations. The major limitation was that, because all the patients were treated with adjuvant FOLFOX, we could not evaluate the interaction between *LINE-1* methylation and treatment effect of adjuvant FOLFOX. Nonetheless, our cohort was relatively homogeneous in stage (stage III and high-risk stages II included) and treatment (surgery and adjuvant FOLFOX at a single institution). Another limitation is the relatively short duration of follow-up. However, the median follow-up duration exceeded 4.5 years, and RFS at which point exhibits a good correlation with longer follow-up periods of overall survival (OS) in colon cancer. Further analyses, including determinations of the OS, will be performed in the future after longer durations of follow-up.

## Conclusions

We found that PS was associated with *LINE-1* methylation in CRC tissue. For stage III colon cancer patients treated with adjuvant FOLFOX, *LINE-1* methylation was associated with RFS. Patients with tumors having concurrent PS(≥1)/*LINE-1*(low) had a higher recurrence rate. Further validation and translational studies to improve treatment outcomes in this subset of patients will be needed in the future. However, *LINE-1* methylation status and combined PS and *LINE-1* statuses were not prognostic parameters in stage III or high-risk stage II CRCs treated with adjuvant FOLFOX.

## Methods

### Patients

A total of 336 CRC patients who received curative surgery and adjuvant chemotherapy in the Seoul National University Hospital, Seoul, Korea, from June 2005 to November 2011 were included. The following eligibility criteria were used for this retrospective study: age at diagnosis > 18 years, stage III (any T and N1 or N2M0) or high-risk stage II (T3 or T4N0M0), completion of at least 6 cycles of adjuvant FOLFOX, and adequate organ functions. High risk was defined as follows: T4 tumor, poor histological grade, lymphovascular invasion, bowel obstruction at presentation, and localized perforation. Exclusion criteria were the following: previous chemotherapy for CRC, previous radiotherapy for CRC, signet ring cell histology, distant metastasis, and history of other malignancies within the previous 5 years. Hereditary non-polyposis colon cancer syndrome or familial adenomatous polyposis was excluded. Patients received either FOLFOX-4 (165 patients) or modified FOLFOX-6 (171 patients). Each cycle of FOLFOX-4 consisted of oxaliplatin (85 mg/m^2^) on day 1 and folinic acid (200 mg/m^2^) and a bolus of 5-FU (400 mg/m^2^) followed by a 22-h infusion of 5-FU (600 mg/m^2^) on days 1 and 2, which was repeated every 2 weeks. Modified FOLFOX-6 consisted of oxaliplatin (85 mg/m^2^), folinic acid (400 mg/m^2^), and a bolus of 5-FU (400 mg/m^2^) followed by a 46-h infusion of 5-FU (2400 mg/m^2^) repeated every 2 weeks. Adjuvant chemotherapy was continued until completion of the planned 12 cycles, recurrence, toxicity, or patient refusal. In 336 patients, 303 (90.2 %) patients had completed all 12 cycles of adjuvant chemotherapy. Thirty-three (9.8 %) patients completed more than 6 cycles but less than 12 cycles of adjuvant chemotherapy. Computed tomography (CT) imaging was performed every six cycles during the chemotherapy period, and patients were followed up at least every 6 months after completion of the chemotherapy. Recurrence was defined based on CT scans in the case of distant metastasis, and the treating oncologist made the decision to perform pathological confirmation when necessary.

The study protocol was reviewed and approved by the institutional review board of Seoul National University Hospital (1408-075-604). Informed consent was exempted because of the retrospective nature of the study and minimal risk of harm to the study subjects. This study was performed in accordance with the recommendations of the Declaration of Helsinki for biomedical research involving human subjects. Patient records/information was anonymized and de-identified prior to analysis.

### Molecular pathologic analyses

Analyses of DNA methylation and microsatellite instability (MSI) were performed as described previously [17, 45]. Briefly, tissue slides were reviewed, and the areas of high tumor cell density (1 cm^2^) were marked and dissected using a knife blade. Normal abdominal LN tissues from the cancer patients were also dissected. Dissected tissues were collected into a microtube containing tissue lysis buffer and proteinase K. The EZ DNA methylation kit was used to convert DNA using sodium bisulfite (Zymo Research, Orange, CA), and *LINE-1* methylation levels were measured using a pyrosequencing methylation assay. We used the same oligonucleotide primers as the ones designed by the Issa group [46]. The primers and PCR conditions are listed in Additional file 3: Table S1. The *LINE-1* assays were performed in a 25-μL PCR reaction containing 2-μL bisulfite-treated genomic DNA, 60 mM Tris-HCl (pH 8.8), 15 mM ammonium sulfate, 0.5 mM MgCl_2_, 1 mM dNTP mix, and 1 U of Taq polymerase. The PCR products were purified and quantified in the PyroMark Q24 System (Biotage AB, Uppsala, Sweden). The amounts of C nucleotides relative to the sum of C and T nucleotides at each CpG site were calculated as percentages. The average of two percentage values in the two adjacent CpG sites (nucleotide positions 321 and 306 of X58075 (GenBank)) was taken as the overall *LINE-1* methylation level in a given sample. The standard deviations for these calculations were given as a supplementary file. For cancer samples, the cut-off values for high and low methylation statuses were set at the median value of *LINE-1* methylation levels in CRCs of PS 0 patients, whereas for lymph node samples, the cut-off values was set at the median value of *LINE-1* methylation levels in LN samples of all the patients.

The MSI status of each tumor was determined using National Cancer Institute’s five consensus microsatellite markers (D2S123, D5S346, D17S250, BAT25, and BAT26). Either the forward or reverse primer for each marker was labeled with fluorescence, and PCR products were electrophoresed and analyzed. We classified MSI status as follows: MSI-high (instability at ≥2 microsatellite markers), MSI-low (instability at 1 marker), or MSS (instability at none).

*BRAF* mutations at codon 600 (V600E) and *KRAS* mutations at codons 12 and 13 were analyzed by a real-time polymerase chain reaction-based allelic discrimination method and direct sequencing, respectively, as previously described [47].

### ECOG-PS measurement

PS was measured at the start of chemotherapy using the ECOG-PS, which is an ordinal scale with scores from 0 to 5 [48]: 0, normal activity; 1, symptomatic but ambulatory; 2, symptomatic—confined to bed/chair <50 % of waking hours; 3, symptomatic—confined to bed/chair >50 % of waking hours; 4, 100 % bedridden; and 5, dead. The present study included 166 cases with PS 0, 169 cases with PS 1, and 1 case with PS 2. For all CRC patients, the PS scores were rated before initiation of adjuvant therapy.

### Statistical analysis

Post hoc exploratory analysis was performed using individual methylation markers. The number of methylation loci was compared using Student’s *t* test or one-way analysis of variance (ANOVA). Categorical variables were compared using chi-square test or Fisher’s exact test. TNR of *LINE-1* methylation level was calculated by the equation (= (average methylation level at *LINE-1* CpG sites 2 and 3 in tumor)/(average methylation level at *LINE-1* CpG sites 2 and 3 in normal LN)). Unconditional logistic regression analysis was performed to measure the association between *LINE-1* methylation and clinicopathological parameters. Clinical data were last updated in November 2014. RFS was calculated from the date of surgery to the date of recurrence or death by the Kaplan-Meier method with log-rank test. To adjust for baseline characteristics, we performed univariate and multivariate analysis using a Cox proportional hazard model. Age, gender, stages (II vs. III), histology (mucinous adenocarcinoma vs. others), tumor location (proximal vs. distal), and MSI status (MSI-high vs. others) were included as covariates. The reason why MSI was put into multivariate analysis include as follows: (1) that MSI is known to be one of the prognostic biomarkers, (2) that MSI status is associated with tumoral *LINE-1* methylation levels [49], and (3) that survival of patients with MSI-high CRCs depends on tumoral LINE-1 methylation status [50]. Two-sided *P* values of <0.05 were considered significant in all analyses. Statistical analyses were performed using SPSS software for Windows, version 17.0 (SPSS, Chicago, IL, USA), but the sample size and power estimation of this study was calculated using PASS software (PASS 08) (NCSS, UT, USA). The Youden index in conjunction with the receiver operating characteristic curve analysis was estimated using SAS software (version 9.3 for Microsoft Windows; SAS Institute, Cary, NC, USA).

## Availability of supporting data

The datasets supporting the results of this article are included within the article.

## Additional files

Additional file 1: Figure S1. Survival analyses using RFS from the date of chemotherapy. (A) Survival analyses show that low *LINE-1* methylation status was closely associated with shorter RFS times (*P* = 0.040). (B) There was no significant difference in RFS according to the performance status (PS). (C) Survival analysis stratified by combinatory PS and *LINE-1*methyaltion statuses. The difference was significant between PS(0)/*LINE-1*(high) and PS(1)/*LINE-1*(low) (*P* = 0.021). (D) Survival analysis using the TNR value in higher BMI score patients. (TIF 3476 kb) Additional file 2: Figure S2. The power and sample size analysis. (A) Overall, 336 subjects (170 in PS ≥ 1 and 166 in PS = 0) achieved 56.93 % power at a 0.050 significance level to detect differences RFS between PS ≥ 1 and PS = 0 groups. (B) The sample size soars as power of ECOG 1 relapse-free proportion increases. (TIF 948 kb) Additional file 3: Table S1. Primer sequences and PCR conditions used for pyrosequencing. (DOC 27 kb)
