# Supplementary figures and images for: Associations and prognostic implications of Eastern Cooperative Oncology Group performance status and tumoral LINE-1 methylation status in stage III colon cancer patients
Source: Clin Epigenetics. 2016 Apr 5;8:36. doi: 10.1186/s13148-016-0203-8 (PMC4820986; doi:10.1186/s13148-016-0203-8)

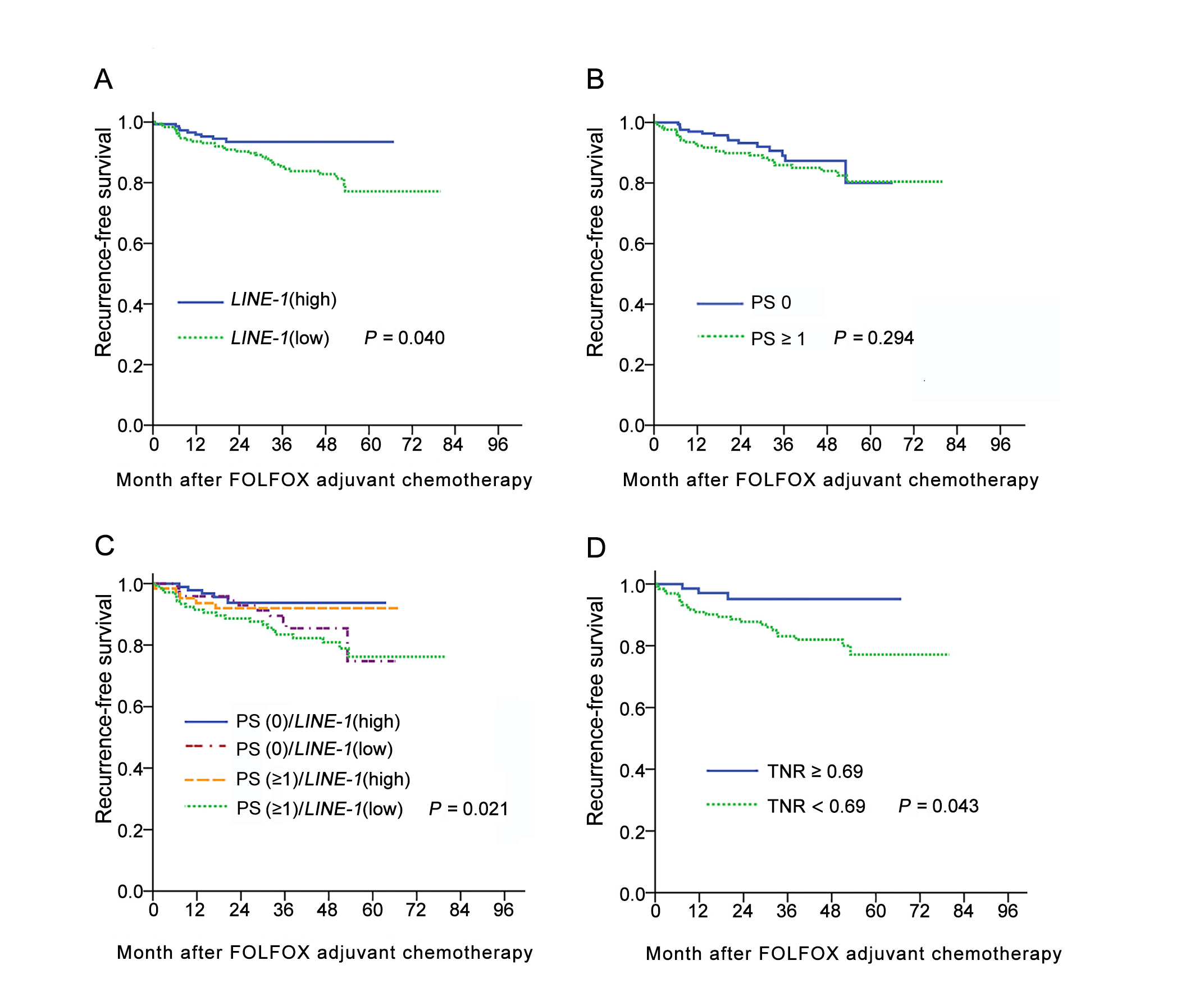

Supplement: Additional file 1: Figure S1. — Survival analyses using RFS from the date of chemotherapy. (A) Survival analyses show that low LINE-1 methylation status was closely associated with shorter RFS times (P = 0.040). (B) There was no significant difference in RFS according to the performance status (PS). (C) Survival analysis stratified by combinatory PS and LINE-1methyaltion statuses. The difference was significant between PS(0)/LINE-1(high) and PS(1)/LINE-1(low) (P = 0.021). (D) Survival analysis using the TNR value in higher BMI score patients. (TIF 3476 kb) [file 13148_2016_203_MOESM1_ESM.tif]

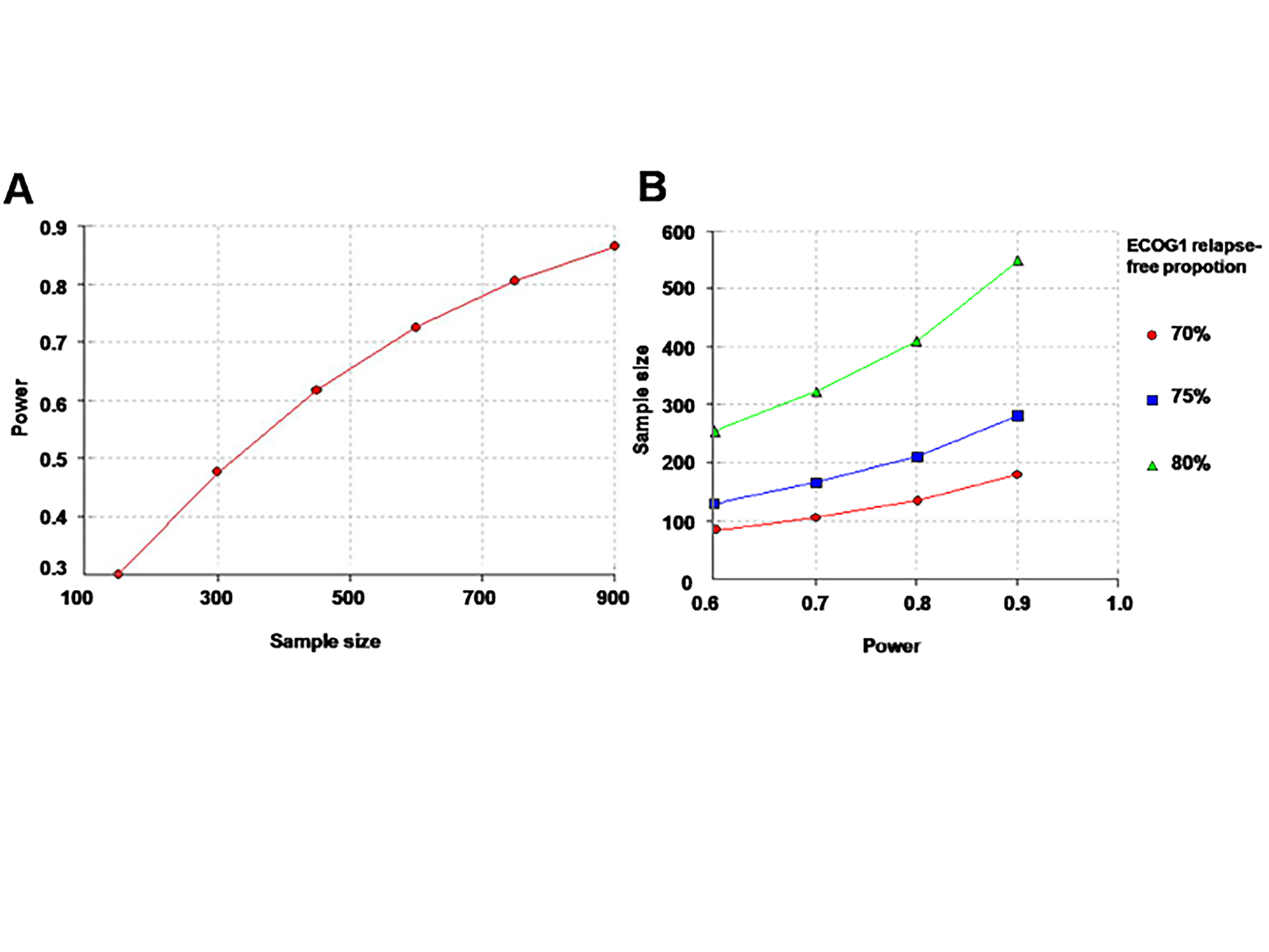

Supplement: Additional file 2: Figure S2. — The power and sample size analysis. (A) Overall, 336 subjects (170 in PS ≥ 1 and 166 in PS = 0) achieved 56.93 % power at a 0.050 significance level to detect differences RFS between PS ≥ 1 and PS = 0 groups. (B) The sample size soars as power of ECOG 1 relapse-free proportion increases. (TIF 948 kb) [file 13148_2016_203_MOESM2_ESM.tif]
