# Supplementary material for: Associations and prognostic implications of Eastern Cooperative Oncology Group performance status and tumoral LINE-1 methylation status in stage III colon cancer patients
Source: Clin Epigenetics. 2016 Apr 5;8:36. doi: 10.1186/s13148-016-0203-8 (PMC4820986; doi:10.1186/s13148-016-0203-8)
Supplement: Additional file 3: Table S1. — Primer sequences and PCR conditions used for pyrosequencing. (DOC 27 kb) [file 13148_2016_203_MOESM3_ESM.doc]

| **Table S1. Primer sequences and PCR conditions used for pyrosequencing** | | |
| --- | --- | --- |
| ***LINE-1*** | **Primer** | **Tm (℃)** |
| **Forward** | 5’-TTTTGAGTTAGGTGTGGGATATA-3’ | **52** |
| **Reverse** | 5’-biotin-AAAATCAAAAAATTCCCTTTC-3’ |
| **Sequencing** | 5’-AGTTAGGTGTGGGATATAGT-3’ |
